# The use of specific coordination behaviours to manage information processing and task distribution in real and simulated trauma teamwork: an observational study

**DOI:** 10.1186/s13049-024-01287-x

**Published:** 2024-12-10

**Authors:** Liselott Fornander, Maria Garrido Granhagen, Ida Molin, Kati Laukkanen, Karin Björnström Karlsson, Peter Berggren, Lena Nilsson

**Affiliations:** 1 Department of Anaesthesiology and Intensive Care, Vrinnevi Hospital, Norrköping, Sweden; 2 Department of Emergency Medicine, Linköping University Hospital, Linköping, Sweden; 3 Department of Anaesthesiology and Intensive Care, Linköping University Hospital, Linköping, Sweden; 4 Department of Biomedical and Clinical Sciences, Linköping University, Linköping, Sweden; 5 Centre for Disaster Medicine and Traumatology, Linköping University, Linköping, Sweden; 6 Department of Computer and Information Science, Linköping University, Linköping, Sweden

**Keywords:** Teamwork, Communication, Simulation validation, Closed-loop communication, Talking to the room

## Abstract

Trauma teams handle severely injured patients under high temporal demands and need to coordinate and achieve collaborative decision-making and task execution through communication. Specific coordination and communication behaviours are taught in simulation training to enhance team performance. An examination of the role and nature of communication could increase the understanding of educational possibilities and assess the validity of in situ simulation on behalf of communication. Our study aim was to describe the relative use of communication within information and task management, the use of coordinating behaviours, and the use of talking to the room and closed-loop communication in in-real-life trauma assessment and in the simulated domain. We video-recorded all verbal communicative events in four real-world trauma teams and four teams during simulation training. The analysis showed that although the teamwork was task-oriented, information management dominated task management at 64% of all the utterances in-real-life and 68% during simulation. In-real-life, information management was dominated by the codes “request information” (24%) and “confirmation” (21%), whereas “task distribution” (43%) was most frequently used for task management. The only difference between domains was that “give information after request” represented a smaller proportion of the utterances in-real-life compared to simulation (*p* ≤ 0.001). Talking to the room was primarily used by the teams in both domains to provide information without request and to delegate tasks. Closed-loop communication was used at a low frequency, 3.6% in-real-life, but was significantly higher, 7.7%, in simulation training. We suspect that this outcome reflects the Hawthorne effect. In the simulations, greater information delivery was provided in response to questions, probably accounting for instructor information. Our results may be valuable for research on trauma team behaviour in a simulated environment to draw conclusions about similar activities in-real-life.

**Supplementary Information:**

The online version contains supplementary material available at 10.1186/s13049-024-01287-x.

## Introduction

Resuscitation of a trauma patient requires a coordinated and collaborative trauma team. Trauma teams are interprofessional and interdisciplinary, often consisting of 7–12 individuals [31, 33]. Trauma teams outside of dedicated trauma centres are often assembled “ad hoc”. The team members coordinate medical information, prioritizations, and taskwork under time pressure due to the urgency of the situation. The work is, to some extent, standardized, but trauma cases are highly variable in their presentation. Trauma teams use verbal communication to handle both task management and information sharing, which potentiates the establishment and coordination of team mental models [14].

There are several specific coordination behaviours to manage information processing and task distribution in trauma teams.

One communication type used is “communication without request”, conceptualized as “online commentaries” [10], “push of information” [14], “provide information without request” (PIWR) [16], and “talking to the room” (TTR) [17, 28]. Another example is the use of “closed-loop communication” (CLC) [23]. CLC means that a team member (sender) clearly makes a call-out, and the receiver verifies the message through a “check-back” that confirms that the information is apprehended correctly. The sender verifies and acknowledges the check-back information [11].

Regarding trauma teams, earlier studies have shown that the in-real-life (IRL) domain gives rise in part to different patterns of communication regarding communication network structures, and linguistic aspects compared to the simulation domain [8, 21]. Such differences might reveal aspects regarding the cognitive fidelity achieved in the simulation domain. The classification system “Coordination System for Medical Teams-Emergency” (CoMet-E) is derived from a system for observing coordination behaviours in anaesthesia teams [19] and has been updated for studying emergency teams [25]. The system does however not separate task- and information related provision of information without request. Schmutz et al. [25] hypothesized that such a distinction could reveal differences between task- and information-related work within different task-types. An information update concerning the patient’s medical history and potential threats correlates with problem solving and decision making, whereas “online commentaries” are used by a team member to brief his/her teammates about steps taken in a task execution. Such measures might help teammates make assumptions about subsequent steps or assistance needed, which can induce back-up behaviour [10] Moreover, CLC is in the CoMet-E system treated as a separate entity, and not categorized to its association with task- or information management.

The rationale behind CLC is to foreclose the possibility of misunderstandings, which in high-hazard environments can have detrimental effects. CLC is today a practised and drilled non-technical skill taught in emergency healthcare simulation and team training [24]. For this reason, it is also used as a measure of team performance [13]. Most studies that explore CLC or use it as an outcome measure conceptualize it as relating to instructions and orders [9, 15]. There is little knowledge about its use in relation to information management [25].

Studies that characterize communication types in medical emergency teams or relate them to results have mostly used experimentally simulated environments [10, 11, 25–28]. To the best of our knowledge, the CoMet-E, has not been used to observe teams in the IRL context. We hypothesised that investigating how the IRL and simulation domains compare regarding verbal communication may verify and update the CoMeT-E classification system in the IRL context and offer specific insight regarding the cognitive fidelity of in-situ simulation of trauma teamwork.

Thus, specific aim of this observational study was to describe the relative use of communication within information and task management, the use of coordinating behaviours, and the use of TTR and CLC in IRL trauma assessment and in the simulated domain.

## Materials and methods

### Procedure and participants

This observational study was set up at the emergency department of a 300-bed regional hospital Vrinnevisjukhuset, in Norrköping, Sweden, which annually received 130–170 trauma patients triaged according to national level 1 trauma criteria. The trauma team was paged to the emergency room (ER) in response to an incoming case. Once every week, identical alerts summoned the team for trauma resuscitation simulation training in the same room. In both situations the specific participants were those in charge and carrying the trauma pager for their role, i.e. randomly chosen. Trained simulation instructors designed and conducted the simulations. They had no role in this research project.

The trauma teams were composed of 9–14 team members. The exact participating roles differed from time to time, but all teams were provided with the following roles:

**Examining physician**, positioned by an ER physician, a surgery resident, or an intern

**Team leader**, positioned by a surgery resident, consultant, or ER physician

**Anaesthesiologist**, positioned by a resident or consultant anaesthesiologist

**Orthopaedic surgeon**, positioned by an orthopaedic surgery resident

**Two ER nurses**, positioned by registered nurses (three years of nursing school), working in the ER

**Airway nurse**, positioned by a nurse with a three-year nursing school and skilled with one year of anaesthesia or intensive care university education

**One or two ER assistant nurse/s**, positioned by ER assistant nurses

In some cases, the following complementary or supporting roles participated during the entire or part of the scenario:

**Intern**, with the role of assisting the examining physician

**Documenting nurse**, positioned by an ER assistant nurse

**One or two instructors,** positioned by ER nurse/assistant nurse/physician with specific instructor training

**Coordinating nurse**, positioned by ER nurses responsible for logistics in the ER

**Consultant anaesthesiologist,** supporting the primary anaesthesiologist

**Retrieving ambulance nurse,** positioned by a nurse with a three-year nursing school and one year of prehospital medicine university education

All potential members of a trauma team were informed through meetings and via email about the study. Informed consent from all active participants was required for the use of the collected video material. The patient was blurred in the editing of the video material. No patient data were filed. Informed patient consent was waived according to the ethical approval of the study (Regional Ethical Review Board in Linköping, Sweden, March 9, 2017 (2017/23–33).

### Measures

#### Data collection

**Fig. 1:**
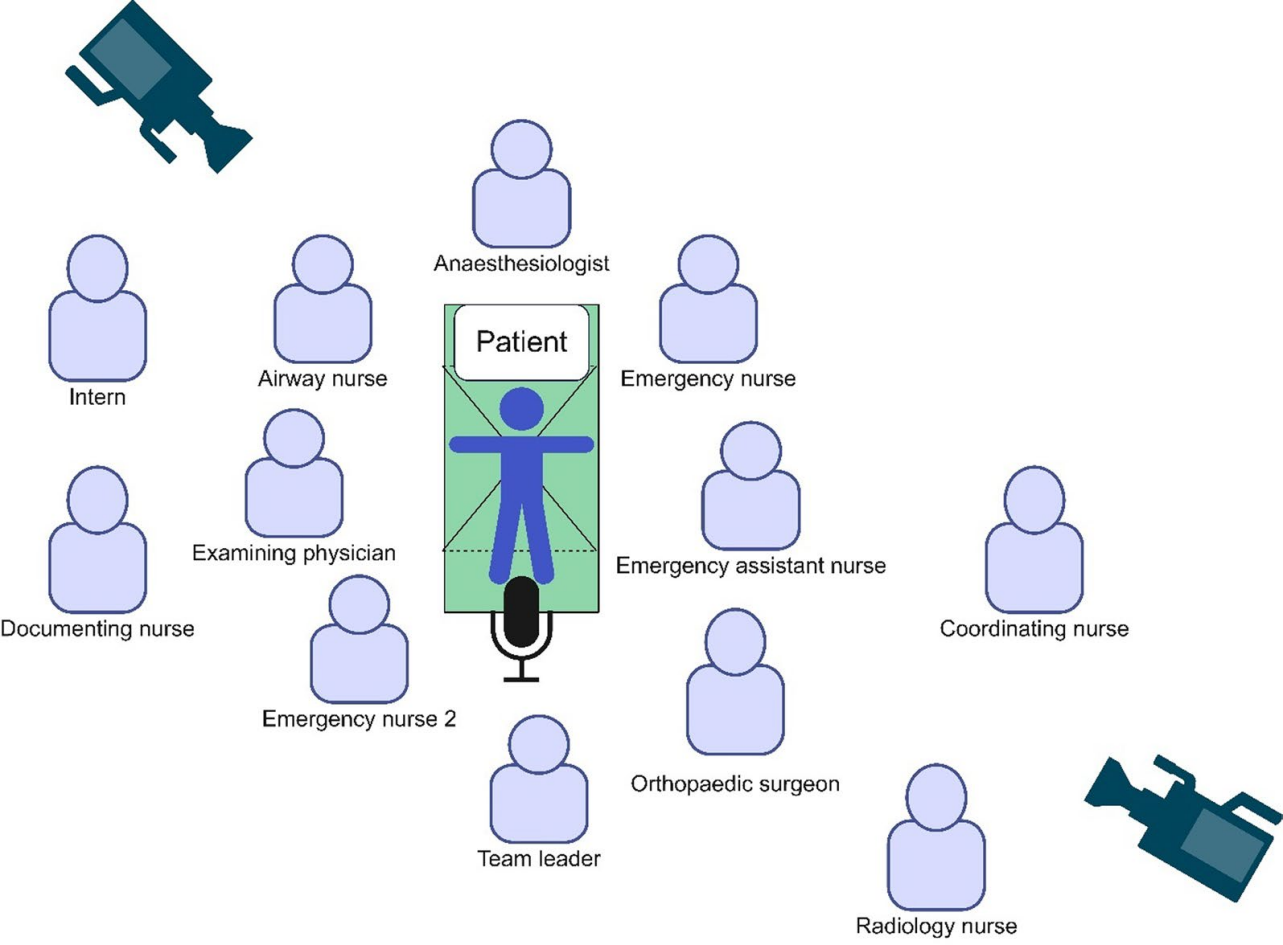
The trauma team, with its members and position of video recording equipment

Two cameras were positioned in the ceiling of the ER (Fig. 1) and controlled from a nearby room. One microphone hung from the roof above the trauma gurney.

During the study period from August 2017 to February 2018, four videos from real trauma resuscitations and four videos from simulated scenarios were included. The same video material was used in another study and was outlined in detail [8]. All cases fulfilled the Swedish National Trauma Triage Criteria [12]. Detailed information about the cases is provided in Table 1.

**Table 1:** Description of the cases handled by teams working in-real-life (IRL) and in simulation (SIM)

Case	Setting	Number of participants in team	Short description
1	IRL	11	Young male, bicycle rider without helmet, high-speed car collision, pain in back and neck, spontaneous nystagmus, stable vital signs
2	IRL	12	Middle-aged man, crashed on a road race bike, amnesia, suspected neurological deficits, stable vital signs
3	IRL	9	Young adult man, knife assault, sharp thoracic laceration wound, respiratory distress
4	IRL	14	Senior male on Warfarin treatment, four-wheel terrain motor vehicle accident, initially unconscious, amnesia, fluctuations in alertness during primary survey, open extremity fracture
5	SIM	11*	Middle-aged woman, squeezed between the wall and an elephant at zoo, declining consciousness, pneumothorax, suspected internal bleeding
6	SIM	12**	Middle-aged woman, self-inflicted fall accident, open bilateral femoral fractures, agitated patient with suspected vessel injury, unstable circulation
7	SIM	12**	Young man, skiing accident, crash against a tree, fractured extremities, declining consciousness
8	SIM	12**	Young woman, horse accident, unstable circulation, suspected pelvic fracture and internal bleeding

*Including one instructor

**Including two instructors

#### Transcription and coding

The verbal communication in the video-recorded material was transcribed verbatim as it occurred temporally. Patient utterances were disregarded. The transcription and coding of the material for the direction of speech has previously been outlined [8]. The transcribed video material was coded based on the instrument CoMeT-E [19, 25] (Supplementary Table A), which descriptively categorizes coordination behaviour. In an earlier study using CoMeT-E, the percentage of time spent on either category was assessed [25], whereas, in this study, all utterances were assessed and coded for categorical belonging.

**Fig. 2:**
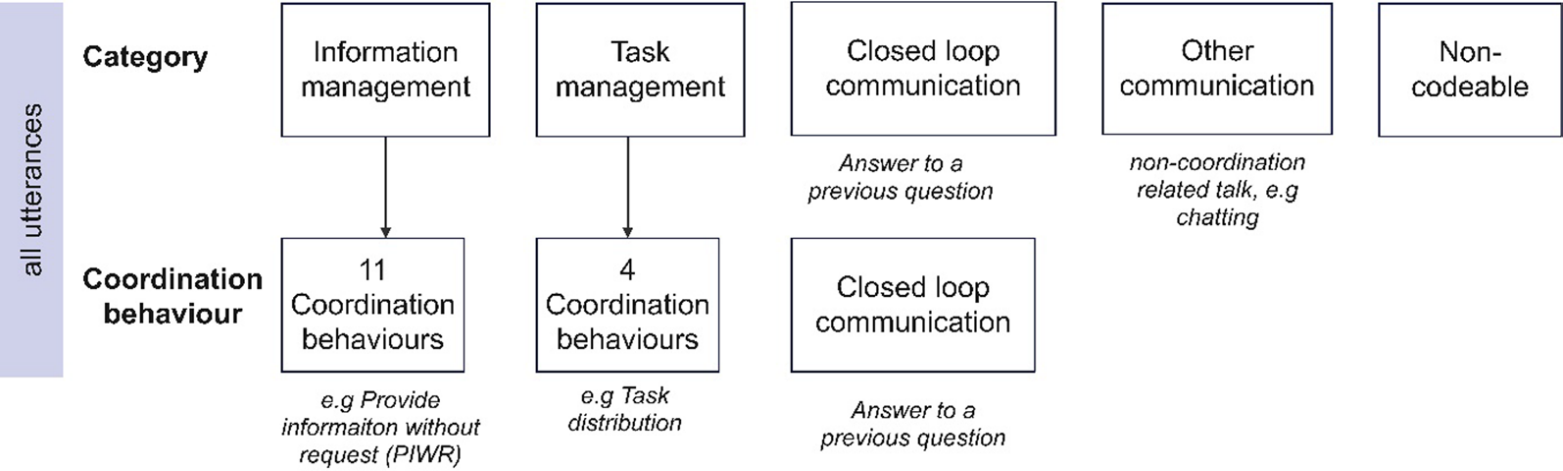
Original Coordination System for Medical Teams-Emergency (CoMeT-E) coding system organization

**Fig. 3:**
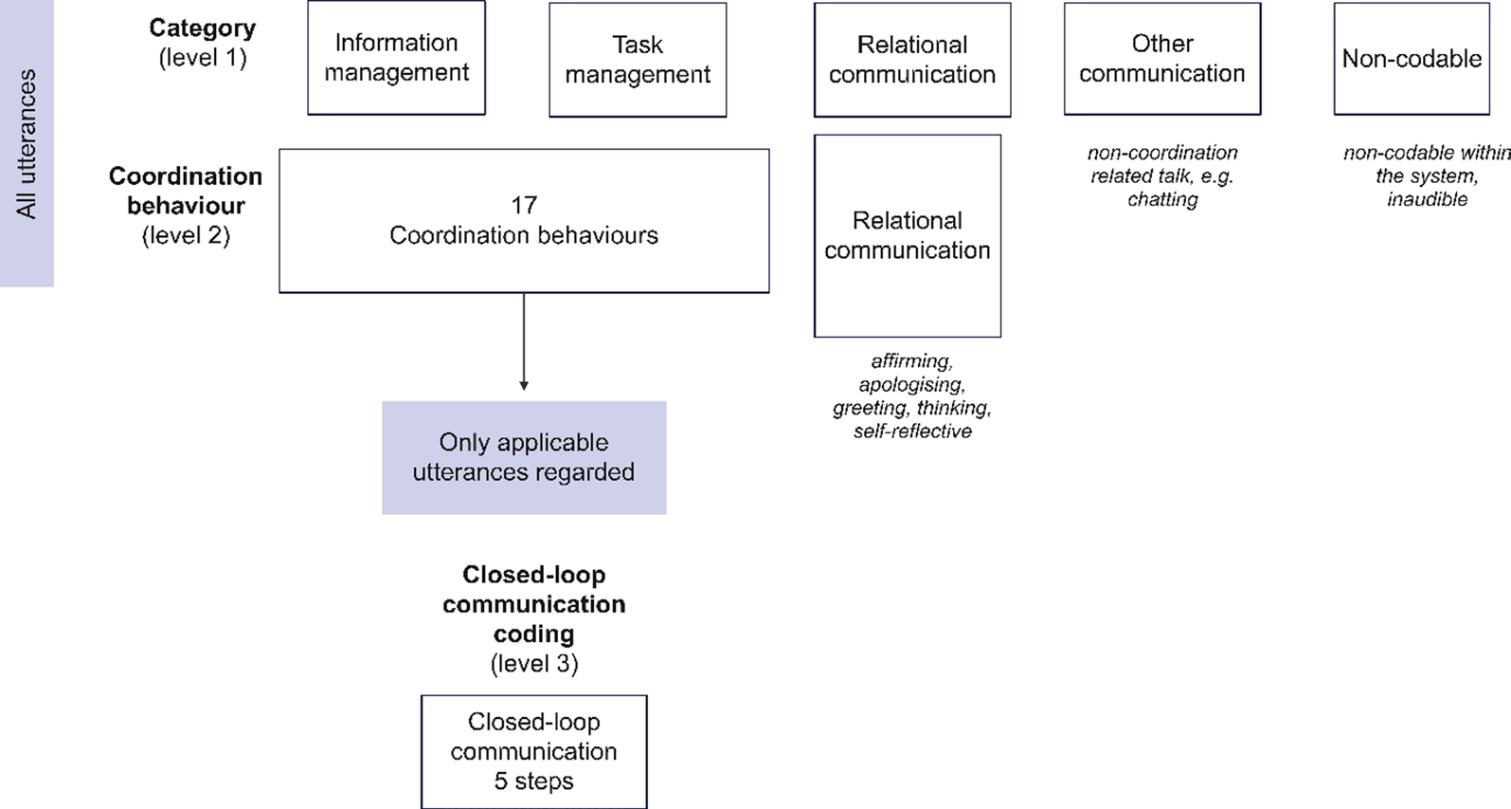
Modified Coordination System for Medical Teams-Emergency (CoMeT-E) coding system organization

To achieve a dynamic coding system, the coordination behaviours, such as “PIWR”, were disengaged from their categories and thereby freely organized into either category. In a previous study, CLC was operationalized as a “check-back” [25] and treated as a separate category. However, since CLC consists of other coordination behaviours, CLC coding was constructed on applicable utterances. The original and modified CoMeT-E coding organizations are visualized in Figs. 2 and 3.

All utterances in the first video (IRL 4) were coded by MGG and LF. Their results were calibrated against a cognition scientist’s (AL) coding of the first half of the same video. Coding differences and difficulties were identified, analyzed, and discussed concerning the three coding levels: level 1, category; level 2, coordination behaviour; and level 3, selected utterances into either of five steps related to CLC.

In response to the material used by the three coders, two new coordination behaviours were added to the system in level 2. These concerned utterances that did not fit into any of the existing coordination behaviours or categories. The added coordination behaviours were “confirmation” and “relational communication”. The latter did not fit into either of the main categories of information- or task-related communication in level 1; thus, a category for relational communication was also added. The modifiedCoMet-E system for categorical coding (level 1) is provided in Table 2. Table 3 displays the modified CoMet-E coordination behaviour coding system (level 2).

**Table 2:** Modified Coordination System for Medical Teams-Emergency (CoMeT-E) main categories (level 1)

Main category	Abbreviation	Contextual information	Example
Information management	IM	Information concerning patient status, diagnoses, the prerequisites of the room, treatment, and other circumstances	“The blood pressure is 140/90”
			“He’s on Warfarin”
			“Is it bleeding, or what do me think?”
Task management	TM	Concerns tasks that are performed or potentially performed, like positioning of the patient	“I am taking the blood pressure now”
			“See if it’s possible to cut the clothes”
			“Who can fetch the ultrasound?”
Relational communication	RCAC	Not related to either information or task management	“Trust the feeling”
			“buy, buy, too bad”
Other communication	OC	Non-coordination related utterances, i.e. chatting	“Hey, what’s the name of the new bar?”
Not codable within the system	U	Inaudible utterances

**Table 3:** Modified Coordination System for Medical Teams-Emergency (CoMeT-E) coordination behaviour (level 2)

Coordination behaviour	Abbreviation	Contextual specification	Example of utterance
Provide information without request	PIWR	No question or request foregoing	“Just to let you know, this patient might have a severe reaction to the anaesthetic that is potentially lethal.”
			“I have turned the nitroglycerin off.”
			“They are pretty much done, take down.”
Request information	RI		“What is the child’s condition?”
			“Do we have a pulse?”
Give information after request	GIAR	Following information request	“Yes, we have a pulse.”
			“The patient’s condition is critical.”
General situation assessment	GSA		“It looks like the things are getting better now.”
Review process	RP		“We checked the pulse, made a blood gas analysis and have an EKG, so everything is done.”
Request team member information	RTMI		“Are you familiar with this equipment?”
Give team member information	GTMI		“I haven’t done this before, but I think I can do it.”
Discuss option	DO		“I am not sure if we can give this medication because of his condition; what do you think?”
Question a decision	QD		“Do you really want me to stop the procedure?”
Evaluate a decision	ED		“I think this was the right choice.”
Make/state a decision	MD		“Let’s forget about this; we need to intubate the patient now.”
Task distribution	TD		“Prepare the adrenaline!”
			“Sue, could you please give me the tube?”
			“Someone come here and give me a hand.”
Planning	PL		“First, we will prepare the medication, then provide the treatment.”
Initiate an action	IA		“Okay, I’ll start now assessing the patient.”
Clarification	CLA		“Just to make sure, you want me to do that?”
Confirmation^a^	C		“Yes, exactly.”
			“We can say that.”
Other communication	OC	Non-coordination related utterance i.e. chatting	“Hey, what is the name of the new bar?”
Relational communication^a^	RCAC		“I feel like I pushed you away now.”
			“We’re the little ones on this side.”
			“Here you go!”
Not codable with the system	U		

^a^Added codes

#### Closed-loop communication

CLC was defined as containing a maximum of five messages, two or more of which had to be present for a CLC to be considered. Only utterances relating to CLC were included in this level 3 coding (Table 4).

**Table 4:** Example of the coding of an utterance

Utterance	Sender	Receiver	Category (level 1)	Coordination behaviour (level 2)	CLC (level 3)
*“Ok, so then, Cefotaxim administered”*	Emergency room nurse	Anaesthe-siologist	Information management	PIWR	Executive confirmation
*“Start from the top”*	Documentary nurse	Assistant nurse 2	Task management	Task distribution	NA
*“I can’t, I cannot reach it”*	Assistant nurse 2	Documen-tary nurse	Task management	Clarification	NA

*CLC* Closed-loop communication; *PIWR* Provide information without request; *NA* not applicable.

To be considered a call-out, a reply to the message was acquired. The framing of CLC was made according to the original conceptualization [11], so that a call-out carries information for another to receive. Hence, a question asked to retrieve information was not considered CLC but could be an antecedent of CLC, for example:

Speaker 1: “I need to know if you are ready”.

Speaker 2: “I am ready”. *Call-out.*

Speaker 1: “OK, you’re ready” *Confirmation.*

However, an instruction framed as a question, for example, “Can you pass the adrenaline?” was considered a call-out if followed by a read-back. To be considered a read-back, the utterance could not only be “yes” or “no”; it had to acknowledge the information part of the call-out. Examples of coding of different sequences are displayed in Table 4.

We maximally used five steps of CLC in order to capture the executive confirmation and check-backs that occur after a task has been completed. This, we argue, is equally important for the team to acknowledge as the check-back confirming the understanding of an order. Not all CLC have five steps, and for information management, two might be sufficient [25]. We therefore regarded CLC as any of the steps, which means at least two steps, since a call-out required a check-back and vice versa.

Subsequent coding and correlation comparison between MGG and LF was made repeatedly until Cohen’s κ = 0.86 was reached (initially 0.64) for the main categories (level 1). After receiving this level of agreement, MGG coded the remaining material. The intercoder agreement of the 20 coordination behaviours of level 2 was also established after discussions and subsequent refinements. When intercoder agreement reached Cohen’s κ = 0.68 (0.54 initially), the coding correlation was deemed satisfactory, and MGG continued individual coding. For level 3, CLC, LF continued the coding when Cohens κ = 0.8 (initially 0.59) was reached. Difficulties in coding were solved through consensus discussions.

#### “Talking to the room” communication

TTR communication was operationalized as *all communication directed to the team*, and further analyzed separately.

### Analytical strategy

The proportion of utterances belonging to the different categories was calculated, and a Chi^2^ test was used to analyse differences between proportional use of coordination behaviour in the IRL and simulated domains. The IBM Statistical Package for the Social Sciences (SPSS) (version 27, Inc., Armonk, NY, USA) was used for all calculations.

## Results

The full material consisted of videos from four IRL and four simulated trauma resuscitations. The total number of utterances amounted to 2476 from the IRL domain and 1728 from the simulated environment. Excellent intercoder agreement was seen for the coding of the main categories as well as for the CLC coding with Cohen’s κ > 0,8, and substantial agreement, with Cohen’s κ 0.61–0.8, was reached for the 19 coordination codes [18].

**Fig. 4:**
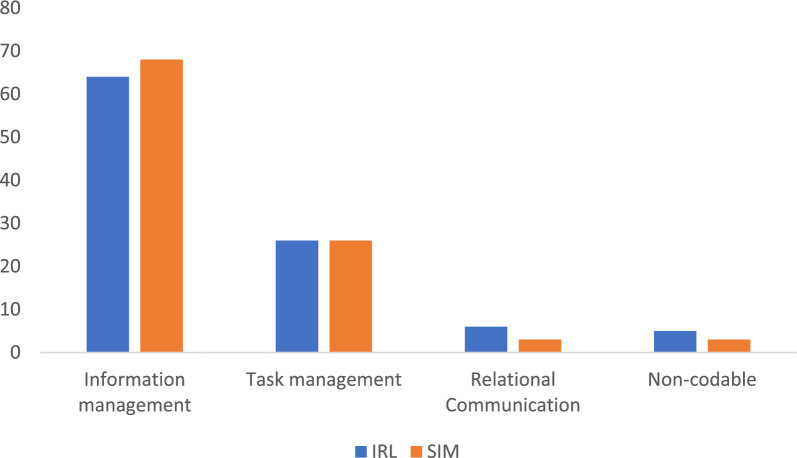
Percentage of utterances belonging to the categories in the in-real-life (IRL) and simulated (SIM) domains

In the IRL domain, 64% of utterances was related to information management and 26% to task management. Relational communication constituted a minor proportion, 6%. When comparing to the simulated domain there was no difference regarding the proportion of information- and task management communication. However, there was significantly more relational communication and non-codable utterances in the IRL compared to the simulated domain. The category “Other communication” was never used and was hence not included in the calculations (Fig. 4).

### Coordination behaviours

**Fig. 5:**
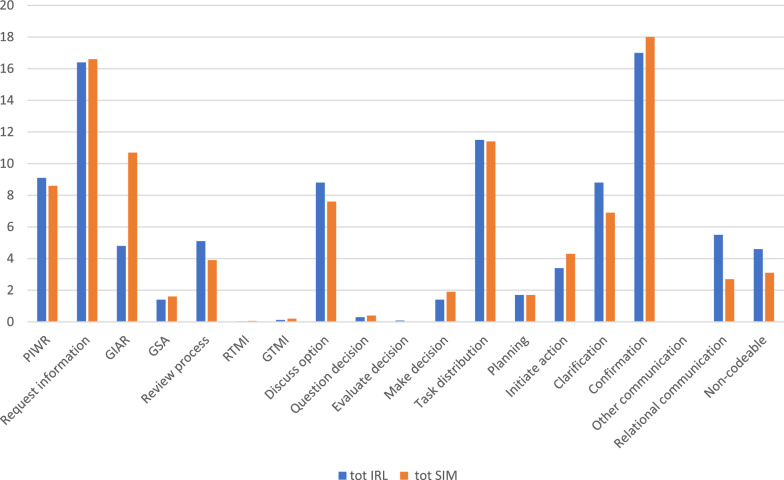
Proportion (%) of the specific coordination behaviours of all utterances in the in-real-life (IRL), blue, and simulated (SIM), red, domains PIWR: Provide information without request. GTMI: Give team member information. RTMI: Request team member information. GSA: General situation assessment. GIAR: Give information after request

The three most used coordination behaviours in the IRL domain were “confirmation”, “request information” and “task distribution” and constituted 17, 16 and 12% of the utterances, respectively. “Provide information without request”, “clarification” and “discuss option” were also frequently used, all around 9% of the total utterances (Fig. 5).

The IRL and simulated domains showed comparable use of coordination behaviours on most parts. However, the code “give information after request” was significantly more prevalent in the simulated domain (*p* ≤ 0.001). In the IRL domain, “relational communication” constituted a significantly greater proportion of the utterances than in simulation (*p* ≤ 0.001) (Fig. 5).

#### Coordination behaviours related to information management and task management

Regarding information management in the IRL domain the codes “request information” (24%), “confirmation” (21%) and “provide information without request” (13%) were the most used. For task management the codes “task distribution” (43%), confirmation” (15%) and “initiate action” (12%) dominated (Table 5).

**Table 5:** Distribution of the most common coordination behaviours used for information and task management

	Domain			
	In real life	Simulation	Total	p-value^a^
Information management				< 0.001
Request information	383 (24)	276 (23)	659 (24)	
Confirmation	324 (21)	245 (21)	569 (21)	
PIWR	208 (13)	139 (12)	347 (13)	
GIAR	110 (7)	178 (15)	288 (10)	
Clarification	173 (11)	101(9)	274 (10)	
Total	1582	1178	1094	
Task management				0.209
Task distribution	278 (43)	190 (42)	468 (43)	
Confirmation	95 (15)	70 (16)	165 (15)	
Initiate action	78 (12)	73 (16)	151 (14)	
Discuss option	50 (8)	38 (8)	88 (8)	
PIWR	18 (3)	10 (2)	28 (3)	
Total	644	450	2760	

Figures denote n (%) of utterances characterized as information and task management; ^a^ Chi-2 test. (Standardized residuals >2/-2 for GIAR)

PIWR: Provide information without request; GIAR: Give information after request.

In comparison between domains, the code “give information after request” represented a smaller proportion of the utterances IRL compared to in simulation (*p* ≤ 0.001). No differences between the IRL and simulated domains were seen regarding task management coordination behaviours (Table 5).

#### Talking to the room

TTR constituted 17% of all utterances in the IRL teams. The coordinating behaviours for information management TTR was dominated by “provide information without request” (20%), “confirmation” (17%) and “review process” (15%) (Table 6), whereas “task distribution” (38%), “initiate action” (18%) and “review process” (15%) were the most frequent coordination behaviours used for task management TTR (Table 7).

**Table 6:** Distribution of the most common coordinating behaviours for information management related to talking to the room (TTR)

Information management talking to the room		
	In real life	Simulation
Coordination behaviour		
PIWR	83 (20)	69 (20)
Confirmation	73 (17)	43 (12)
Review process	61 (15)	*38 (11)*
GSA	31 (7)	24 (7)
Request information	*56 (13)*	64 (18)

Figures denote n (%) of utterances characterized as information management talking to the room PIWR: provide information without request; GSA: General situation assessment. Coordination behaviours that were not dominant but presented to illustrate differences are presented in italics.

**Table 7:** Distribution of the most common coordinating behaviours for related task management related TTR

Task management talking to the room		
	In real life	Simulation
Coordination behaviour		
Task distribution	77 (38)	67 (41)
Initiate action	37 (18)	47 (28)
Confirmation	26 (13)	*6 (4)*
Discuss option	10 (5)	14 (8)
PIWR	*3 (1)*	*2 (1)*

Figures denote n (%) of utterances characterized task management talking to the room; *PIWR* provide information without request. Coordination behaviours that were not dominant but presented to illustrate differences are presented in italics.

In the simulated teams, TTR constituted 20% of the utterances. Similar to IRL, PIWR dominated information management TTR, however “request information” was the second most frequent TTR in the simulated domain (Table 6). Regarding task management, “confirmation” was more frequently used in the IRL scenario, whereas “initiate action” and “discuss option” were more prominent during the simulation (Table 7).

#### Closed-loop communication

The use of CLC was coupled to to 3.6% of all utterances in the IRL domain. CLC in connection with information management was used in 4.8% of utterances and task management in 2.0% (Table 8).

**Table 8:** Proportion of utterances coupled to closed-loop communication in real life and simulated domains

	Domain			
	In real life	Simulation	Total	*p*-value^a^
Closed-loop communication, any part, n (%)	89 (3.6)	133 (7.7)	222 (5.3)	< 0.001
Total, n	2477	1728	4205	
Information management, n (%)	76 (4.8)	89 (7.6)	165 (6.0)	0.003
Total, n	1583	1178	2761	
Task management, n (%)	13 (2.0)	43 (9.6)	56 (5.1)	< 0.001
Total, n	644	450	1094	
Relational communication, n (%)	0 (0)	1 (0.7)	1 (0.3)	n.v
Total, n	250	100	350	

When comparing the different domains, CLC was significantly more prevalent in the simulated domain than in IRL (p ≤ 0.001; in 7.7% and 3.6% of all utterances, respectively) (Table 8).

Both task- and information management-related CLC shared this significant distribution difference.

**Fig. 6:**
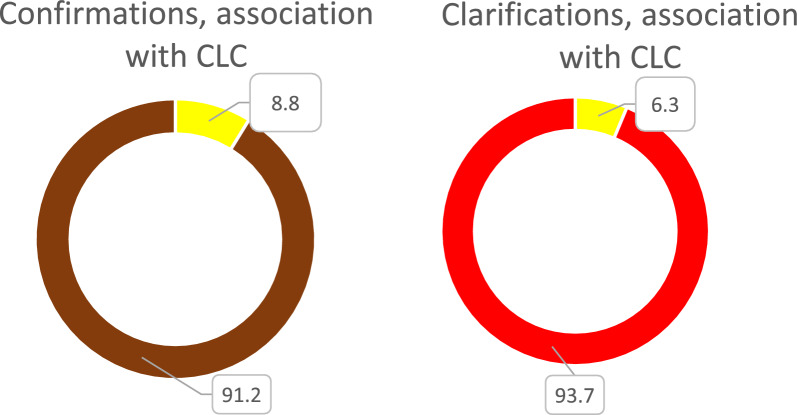
Proportion of Confirmations and Clarifications associated with Closed loop communication (CLC)(any part), is displayed in yellow of the respective coordination behaviours Confirmations (brown circle) and Clarifications (red circle)

The coordination behaviours Confirmation and Clarification, accounting for 17,5% and 8,8% of all utterances of the IRL and the simulated cases were associated with CLC in 8,8% and 6%, respectively (Fig. 6).

## Discussion

This study assessed coordination behaviours during trauma teamwork IRL and during simulation. It showed a similar distribution of utterances related to task and information management in both domains, with confirmations being the most common coordination behaviour. Detailed knowledge about how these domains compare adds information that can be used to present a validity argument for trauma communication research in simulation. This is valuable since conclusions about behaviour in the IRL are drawn upon behaviour in the simulated environment. Further, coordinating behaviours and communication types have been classified in teamwork from different domains of healthcare and other organizations. These have been confirmed and validated in emergency teams [10, 25] and trauma teams [2, 11, 14]. The present study inquired a more detailed description about the relevance in this specific domain. This might be informative to simulation based training, as specific patterns of communication can be linked to the specificities of the trauma environment.

### Information and task management

The trauma team is often described as performing task-oriented teamwork, and the pace is high. There are working manuals and a specific order of patient examination that structure the work performed. Although task-focused in character, these teams’ communication is largely represented by information processing (65%). The “push of information” is considered positive for team performance [14, 22, 32], and this type of coordination behaviour is called “PIWR” in the CoMeT-E system. It functions to update team members about particular information that is, by the sender, believed important for the receiver without carrying out specific guidance, instructions, or questions.

We found, however, that explicit information management through “request information” and “confirmation” constituted larger proportions of the information management activity, each comprising 20% of the information related communication, whereas the implicit information related behaviour “PIWR” amounted to just below 10%.

One important difference between the IRL and simulation domains was that “give information after request” was significantly more prevalent during simulation. This is most reasonably the result of instructor delivery of information upon request from the team members. Although in theory, this might reduce the need for “PIWR” in simulation (as team members get information updates through such common information exchange), we did not find any significant reduction in “PIWR”.

Overall, the proportions of task management coordination behaviours were similar between the two domains. The two most important task management coordination types were “task distribution”, accounting for as much as 43%, and “confirmation”. Explicit task distribution is generally considered positive for accomplishing tasks in emergency teams [5, 7, 19]. In the present study, earlier described online commentaries, which are comments about own activities and task executions, were probably comprised of both “PIWR” and “initiate action”, depending on the exact spell-out. Such comments are often more implicit and aim to make others aware of steps taken or planned in a process that could possibly induce back-up behaviours [10, 16]. Task-related communication in the form of task distribution and instruction is more beneficial to performance in heterogeneous teams, which, to a greater extent, need explicit instructions. An experienced and homogeneous team might benefit from reducing task-related communication, as overt communication slows down task execution and makes less room for information processing and decision-making processes [16, 25]. Technical skills training with a honing of tasks is a pillar of trauma team training [6], and even though such training is technical in nature, it could supposedly affect communication in a positive way by reducing explicit task-related communication.

### Talking to the room

The main purpose of TTR communication is to deliver important information updates with the aim of aligning mental models within the team. In earlier studies, this has been conceptualized as either TTR, “push of information”, or PIWR [14, 15, 32]. We considered all utterances directed to the team in this study and found that overall in both domains, around 20% of the information-related TTR communication was “PIWR”, thus representing the first level of information sharing to the team (fact sharing) [30]. Such communication has been linked to improved performance in clinical diagnostic processes [29]. However, such an outcome could not be demonstrated in emergency resuscitation scenarios [25, 29].

“Review process” and “general situation assessment”, representing the provision of interpretation of facts and possibly projection sharing, were less frequent in this study, both IRL and during simulation. These types of communication have been associated with the alignment of mental models. Fact sharing might result in the oversharing of basic facts that are already known to the other team members, and thereby carries the risk of contributing to information overload (Sohrab et al. 2015). This tendency might, to a great extent, result from the trauma team handling algorithm-like processes in which background assumptions and representations are alike. Therefore, the sharing of basic facts is not merely a sharing of facts but a triggering of similar mental processes across individuals. An example of this is the subtle expression of concern by repeating a fact about a patient, such as “the systolic blood pressure is at 80”, with the implicit intention of pointing at the need to address this concern in action [3]. Such commentaries serve to decrease potential attention narrowing in other team members, and are fundamental to safe teamwork [4]. With a similar frame of reference, the need to share interpretation and mental projections represented by the “review process” and “general situation assessment” is reduced. Future studies could test the hypothesis that team mental models can be updated and corrected through the use of PIWR in algorithm-like processes, whereas “review process” and “general situation assessment” are required in knowledge-related processes.

Information related TTR were overall more explicit than expected, with “confirmation” being an important coordination behaviour in both domains. However, “request of information” was one of the most important information related TTR in simulation, but not used to the same extent IRL. Overall, the findings imply that besides providing a push of information and assessments, a central communicator also addresses information gaps and acknowledges others. Both of these coordination behaviours represent TTR that is more explicit in nature than earlier conceptualizations of information-related TTR.

Task management TTR in both domains mainly regarded “task distribution” and the “initiation of actions”. The initiation of actions delivered to the team probably functions as the previously described concept of online commentaries—that is, to update the team on steps taken, which stimulates implicit actions and back-up behaviour [10]. However, regular task distributions dominated the “initiation of actions” regarding TTR, which underlines the need for a central speaker to explicitly instruct, delegate, and coordinate task executions in a trauma team and underlines team member interdependence.

### Closed-loop communication

CLC is considered an important communication tool that is used in fast-paced team practice to acknowledge correct information transfer and the execution of an instruction. In our conceptualization of CLC, we excluded question–answer communication as well as possible call-outs in which no acknowledgement happened or the reception part consisted of an acknowledgement (confirmation) without a check-back of the call-out information. We included check-backs and acknowledgements occurring subsequent to the execution of tasks as they fit the CLC structure. We looked for the use of CLC and detected it as any part of CLC that ultimately required at least two steps of the original description. Surprisingly, we found a small proportion of the utterances was associated with CLC, and significantly less used IRL. The proportional use of CLC is not easily compared to previous studies, since in most cases, CLC has been counted and reported as a number per team, or the frequency of completed check-backs to observed call-outs, without relating it to the bulk of communication.

In this study, the CoMeT-E system was modified according to the material and the research questions. One of the two added codes for coordination behaviour was “confirmation”, which proved to be one of the most prevalent ones. Sometimes, the confirmation was part of a CLC, such as a “check-back”, but in most cases, it was provided as “yes” or “OK” and was not associated with CLC. This leads us to the conclusion that information and task management are in many instances acknowledged in ways other than through check-backs, perhaps at the expense of repetition and thereby precision but with the possibility of reducing communication load.

In a sociolinguistic study of obstetric teams, the best-performing teams oriented towards CLC but with shorter linguistic structures compared to the textbook variant [1]. Some scholars have argued that the ritualization of speech ignores the dynamics of interaction and the multiple ways in which a message is best delivered [1], and the findings from our study support the suspicion that real-life CLC might differ from textbook CLC and that firm ritualization might increase communicative load at the expense of efficiency [25].

Further, we expected the use of CLC would be coupled to task management, but in this study, CLC was a greater part of information management communication in the IRL domain, and task management in the simulated domain. It is reasonable to believe that more tasks and administrations were executed in the simulation domain, since the simulations were created to induce rapid decision-making and resuscitation task coordination. In a study assessing CLC use in IRL trauma teams, the use of CLC was more frequent in the most serious cases [2]. The use of check-backs also seems to be more frequent in response to medication orders than intravenous fluid administration orders or task delegations [2, 9]. In our study, besides the task-related CLC, the overall task-related communication was similar to IRL, but there was also significantly higher CLC use regarding information management in simulation. This suggests the presence of at least some level of a Hawthorne effect, i.e. the knowledge of being watched and probably assessed on behalf of communication increases the use of “desired communication” [20]. The high frequency of confirmations and clarifications unrelated to CLC suggests that for many call-outs other ways to confirm the sender message are used.

### Methodological considerations

This study is strengthened by the comprehensiveness of the description of the communication used, and the use of both the IRL and the in-situ simulated environment.

The weaknesses include the use of a single center material, which might limit the generalizability, and although rich in verbal communicative events, a limited number of studied cases. Although all teams were composed from the daily schedules, we have not assessed possible interpersonal relationships in the different teams, or the possible impact of different contextual conditions. Treating qualitative data, such as communication, quantitatively will not be completely explanatory, as communication is dynamic, and situationally coupled. Although the quantifications provide some information, they do not reveal other important aspects that are relevant for the understanding of communication, and which can be addressed through pure qualitative inquiries that carry the possibility to penetrate deep into a smaller sub-set of situations. Examples of this are particularities of the case, the training and experiences of the team members, and other situational factors applied to the timing and setting of each trauma resuscitation. Further, this study did not address the quality of clinical practice in the trauma teams including the relationship between communication and medical action undertaken. The quantifications and comparisons of verbal utterances collected from different trauma cases, handled by different teams might bias the results, and a comparison simply based on the IRL or simulation domains might obscure other important situational factors. However, e.g. Gundrosen et al. [10], qualitatively studied team talk in simulation, and opened for the possibility that the patterns seen in simulation might be specifically shaped by the use of a dummy and an instructor providing the “missing cues”. This might affect team talk patterns, based on the environment regardless of particularities of the case. From our results we have shed further light on this important educational issue.

A main methodological consideration regards our use of the CoMeT-E system for the classification of coordination behaviours. The acquisition of a comprehensive coding system was important for a truthful analysis of our material, as we aimed to quantitatively describe and compare the use of speech in different domains. The CoMeT-E system was modified to fit the material and the research aim, and TTR was operationalized as all verbal communication directed to the team. Thus, the coding system was complemented with a specific code and category relating to relational talk (e.g. greetings and salutations). Further, confirmations were added as a separate coordination behaviour to achieve an exhaustive coding scheme. CLC was originally considered a coordination behaviour distinct from the categories of information and task management. A strength is our separate coding of CLC to explain its association with task and information management and its association with specific coordination behaviours. As we did not include call-outs that were never responded to, no data about “missed” confirmations or check-backs was collected.

Substantial work was executed to frame the codes according to their meaning, thereby increasing strict and coherent coding. In this study, the coordination behaviours “request team member information”, “give team member information”, and “evaluate decision” were used at a minimum, which disqualified any conclusions drawn from those observations. The original CoMeT-E system would benefit from reducing alternative coding of behaviours associated with decision-making from three to one to achieve a useful instrument. For many purposes, it would also be serviceable to coalesce codes in which retrieval and giving of information and team information are separated into “information retrieval” and “team member information retrieval”.

We coded all utterances except for patient utterances, which were left out for ethical reasons. This might have somewhat skewed the IRL utterances. It is not typically for patients to talk a lot during resuscitation, but a less injured patient can be interviewed and provide information to the team.

## Conclusion

The modified CoMeT-E showed serviceable to describe the nature of utterances relative to coordination both in simulated and real-life trauma teams. We found that information management dominated the teamwork. TTR information management was mostly implicit with PIWR being the most important strategy. TTR less frequently revealed situation updates and served interpretations to the team. This suggests a team with a similar frame of reference and mental models, leaning on team members with a possibility to individually integrate and interpret the incoming information. When TTR was used in relation to task management, it was more explicit in nature and most often concerned task distribution.

The comparison of IRL and In-situ simulation of trauma teamwork revealed overall a similar relative distribution of coordination behaviours. However, in the simulated domain the communication towards, and from the instructor might have altered the pattern somewhat, as we saw a greater use of “give information after request”. CLC was used to a greater extent in the simulation domain, which could result from a higher resuscitation intensity or represent a Hawthorne effect. In either case, it is worth noting that CLC use was low in relation to all verbal communication taking place in the emergency room. Perhaps this illustrates that CLC is a tool that is predominantly used to convey and check-back the most important messages. In a physically assembled team non-verbal communication probably substitute for check-backs in some cases, which seems rational, since information overload presents a threat to team functioning.

## Supplementary Information

Supplementary file 1

## Data Availability

Data sharing of original videos is not possible, as they are sensitive in nature. The datasets supporting the findings of this article are available from the corresponding author, LF, upon reasonable request.
