# Supplementary material for: The use of specific coordination behaviours to manage information processing and task distribution in real and simulated trauma teamwork: an observational study
Source: Scand J Trauma Resusc Emerg Med. 2024 Dec 10;32:128. doi: 10.1186/s13049-024-01287-x (PMC11629511; doi:10.1186/s13049-024-01287-x)
Supplement: Supplementary file 1 [file 13049_2024_1287_MOESM1_ESM.docx]

# **Appendices**

Supplementary Table A.

| CoMeT-E observation categories and codes | | |  |
| --- | --- | --- | --- |
| **Main category** | **Coordination code** | **Contextual specification** | **Example** |
|  |  |  |  |
| Closed-loop communication | Closed-loop communication | Answer to a  previous code | “Yes, I will prepare the heparin.” |
|  |  |  | “Okay, will do that.” |
|  |  |  | “Yes, understood.” |
|  |  |  | “No, please repeat.” |
| Information | Provide information without  Request | No question or  request foregoing | “Just to let you know, this patient might have a severe reaction to the anaesthetic that is potentially lethal.” |
|  |  |  | “I have turned the nitroglycerin off.” |
|  |  |  | “They are pretty much done, take down.” |
|  | Request information |  | “What is the child’s condition?” |
|  |  |  | “Do we have a pulse?” |
|  | Give information after request | Following information  request | “Yes, we have a pulse.” |
|  |  |  | “The patient’s condition is critical.” (reply after a question) |
|  | General situation assessment |  | “It looks like the things are getting better now.” |
|  | Review process |  | “We checked the pulse, made a blood gas analysis and have an EKG, so everything is done.” |
|  | Request team member information |  | “Are you familiar with this equipment?” |
|  | Give team member information |  | “I haven’t done this before, but I think I can do it.” |
|  | Discuss option |  | “I am not sure if we can give this medication  because of his condition; what do you think?” |
|  | Question a decision |  | “Do you really want me to stop the procedure?” |
|  | Evaluate a decision |  | “I think this was the right choice.” |
|  | Make/state a decision |  | “Let’s forget about this; we need to intubate the patient now.” |
| Task management | Task distribution |  | “Prepare the adrenaline!” |
|  |  |  | “Sue, could you please give me the tubas?” |
|  |  |  | “Someone come here and give me a hand.” |
|  | Planning |  | “First, we will prepare the medication, then provide the treatment.” |
|  | Initiate an action |  | “Okay, I’ll start now assessing the patient.” |
|  | Clarification |  | “Just to make sure, you want me to do that?” |
| Other communication | Non-coordination related utterance, i.e. chatting |  | ”Hey, what is the name of the new bar?” |
| Non-codable utterance | All utterances not codable with the system | | |
